# Lactoferrin Against SARS-CoV-2: *In Vitro* and *In Silico* Evidences

**DOI:** 10.3389/fphar.2021.666600

**Published:** 2021-06-17

**Authors:** Elena Campione, Caterina Lanna, Terenzio Cosio, Luigi Rosa, Maria Pia Conte, Federico Iacovelli, Alice Romeo, Mattia Falconi, Claudia Del Vecchio, Elisa Franchin, Maria Stella Lia, Marilena Minieri, Carlo Chiaramonte, Marco Ciotti, Marzia Nuccetelli, Alessandro Terrinoni, Ilaria Iannuzzi, Luca Coppeda, Andrea Magrini, Sergio Bernardini, Stefano Sabatini, Felice Rosapepe, Pier Luigi Bartoletti, Nicola Moricca, Andrea Di Lorenzo, Massimo Andreoni, Loredana Sarmati, Alessandro Miani, Prisco Piscitelli, Piera Valenti, Luca Bianchi

**Affiliations:** ^1^ Dermatology Unit, Department of Systems Medicine, Tor Vergata University Hospital, Rome, Italy; ^2^ Department of Public Health and Infectious Diseases, University of Rome “La Sapienza”, Rome, Italy; ^3^ Department of Biology, Structural Bioinformatics Group, University of Rome “Tor Vergata”, Rome, Italy; ^4^ Department of Molecular Medicine, University of Padova, Padova, Italy; ^5^ Department of Experimental Medicine, Tor Vergata University Hospital, Rome, Italy; ^6^ Department of Statistics, University of Rome Tor Vergata, Rome, Italy; ^7^ Virology Unit, Tor Vergata University Hospital, Rome, Italy; ^8^ Laboratory Medicine, Department of Experimental Medicine and Surgery, Tor Vergata University Hospital, Rome, Italy; ^9^ Occupational Medicine Department, University of Rome “Tor Vergata”, Rome, Italy; ^10^ Villa dei Pini Hospital, Anzio, Italy; ^11^ Pineta Grande Hospital, Caserta, Italy; ^12^ Fimmg provincial, Rome, Italy; ^13^ Infectious Disease Unit, Tor Vergata University Hospital, Rome, Italy; ^14^ Department of Environmental Sciences and Policy, University of Milan, Milan, Italy; ^15^ UNESCO Chair on Health Education and Sustainable Development, University of Naples Federico II, Naples, Italy

**Keywords:** lactoferrin, COVID-19, SARS-CoV-2, spike, bovine lactoferrin

## Abstract

Lactoferrin (Lf) is a cationic glycoprotein synthetized by exocrine glands and is present in all human secretions. It is also secreted by neutrophils in infection and inflammation sites. This glycoprotein possesses antimicrobial activity due to its capability to chelate two ferric ions per molecule, as well as to interact with bacterial and viral anionic surface components. The cationic features of Lf bind to cells, protecting the host from bacterial and viral injuries. Its anti-inflammatory activity is mediated by the ability to enter inside the nucleus of host cells, thus inhibiting the synthesis of proinflammatory cytokine genes. In particular, Lf down-regulates the synthesis of IL-6, which is involved in iron homeostasis disorders and leads to intracellular iron overload, favoring viral replication and infection. The well-known antiviral activity of Lf has been demonstrated against DNA, RNA, and enveloped and naked viruses and, therefore, Lf could be efficient in counteracting also SARS-CoV-2 infection. For this purpose, we performed *in vitro* assays, proving that Lf exerts an antiviral activity against SARS-COV-2 through direct attachment to both SARS-CoV-2 and cell surface components. This activity varied according to concentration (100/500 μg/ml), multiplicity of infection (0.1/0.01), and cell type (Vero E6/Caco-2 cells). Interestingly, the *in silico* results strongly supported the hypothesis of a direct recognition between Lf and the spike S glycoprotein, which can thus hinder viral entry into the cells. These *in vitro* observations led us to speculate a potential supplementary role of Lf in the management of COVID-19 patients.

## Introduction

In December 2019, in Wuhan, China, a cluster of pneumonia cases was observed. This cluster was related to a novel member of *Betacoronavirus*, named SARS-CoV-2, possessing more than 80% identity to SARS-CoV and 50% to the MERS-CoV (Lu et al., 2020; Tian et al., 2020). Coronavirus are spherical, enveloped viruses possessing a single-stranded, positive-sense RNA genome with a length between 26 and 32 kilobases (Su et al., 2016). Their genome encodes 16 nonstructural proteins (Menachery et al., 2014), accessory proteins (Forni et al., 2017), and four fundamental structural proteins, specifically spike (S) glycoprotein, small envelope protein, matrix protein, and nucleocapsid protein (Lan et al., 2020). Homotrimeric S glycoprotein, possessing N-linked glycans, is located on the envelope and comprises two functional subunits (S1 and S2) in each spike monomer (Cui et al., 2019). As homotrimers of S glycoproteins are exposed on the viral surface, they are involved in both host receptors binding (S1) and membrane fusion (S2) (Li, 2016; Lu et al., 2020). Cryo-electron microscopy (cryo-EM) has highlighted S protein interactions with the cell receptor angiotensin-converting enzyme 2 (ACE2) and the dissociation of the S1 domain after its binding to the host cells, which leads to the S2 domain transition to a more stable conformational state, pivotal for membrane fusion (Gui et al., 2017; Yuan et al., 2017; Kirchdoerfer et al., 2018). Apart from ACE2, the heparan sulfate proteoglycans (HSPGs) located on the cell surface have been recognized as other binding sites for SARS-CoV (Lang et al., 2011) and could be important also for SARS-CoV-2 in the early attachment phase.

Lately, Wrapp et al. (Wrapp et al., 2020), identified the first 3.5-Å-resolution cryo-EM structure of the SARS-CoV-2 S trimer in the prefusion conformation. Because of its critical function in the SARS-CoV-2 infection course, the S glycoprotein is a target for antibody, protein, and drug-mediated neutralization, and the understanding of its three-dimensional structure allowed us to get atomic-level information essential for the design and development of innovative therapeutic molecules (Romeo et al., 2020).

Considering the hypothesis that innate immunity could suggest possible molecules with antiviral activity against SARS-CoV-2, we highlighted how children, in which innate immunity is more prominent (Lang and Zhao, 2020), are less likely to suffer from severe or critical COVID-19 disease compared to adults (Rosa et al., 2017; Woodman et al., 2018). Indeed, preliminary evidences suggested that the breast milk isolated from positive COVID-19 mothers does not contain SARS-CoV-2 particles (Lang and Zhao, 2020).

Lactoferrin (Lf) is a glycoprotein of the transferrin family possessing several functions (Valenti and Antonini, 2005; Rosa et al., 2017). It is synthetized by exocrine glands and neutrophils and is present in human milk and in all secretions (Valenti and Antonini, 2005; Rosa et al., 2017). Since this protein is one of the most important factors of innate immunity, constituting a well-known barrier against pathogens colonizing both mother and fetal habitats (Woodman et al., 2018), it can be hypothesized that it could also act as a potential nutraceutical agent capable of contrasting SARS-CoV-2 infection.

Indeed, two promising in vitro studies on SARS-CoV (Lang et al., 2011) and on SARS-CoV-2 (Hu et al., 2021) have demonstrated that Lf inhibits the early phase of virus infection.

Lf has four pleiotropic activities: chelation of two ferric ions per molecule, interaction with anionic compounds, translocation into the nucleus and modulation of inflammation and iron homeostasis. Lf capability to chelate two ferric ions per molecule influences bacterial and viral replication and hinders reactive oxygen species formation (Valenti and Antonini, 2005; Berlutti et al., 2011; Wakabayashi et al., 2014). The binding of Lf to anionic surface components, thanks to its cationic features, is associated with the host protection against bacterial and viral adhesion and entry (Valenti and Antonini, 2005). Moreover, the entrance of Lf into host cells and its translocation into the nucleus (Ashida et al., 2004; Lepanto et al., 2019) is related to its anti-inflammatory function (Suzuki et al., 2008; Liao et al., 2012; Kruzel et al., 2017). Furthermore, Lf ability to restore iron homeostasis, perturbed by viral infection and inflammation (Mancinelli et al., 2020), is associated with its ability to chelate iron, decrease iron overload, diminish IL-6 levels, and modulate iron proteins. Iron homeostasis is guaranteed by the expression of some iron proteins such as transferrin, ferroportin, hepcidin, and ferritin. The disorders of iron homeostasis, induced by inflammation, increase intracellular iron concentration, thus favoring viral replication (Campione et al., 2020). Moreover, Lf seems to modulate the plasminogen activation and control the coagulation cascade with a remarkable antithrombotic activity (Zwirzitz et al., 2018), a very frequent complication of SARS-CoV-2 infection (Marietta et al., 2020). In addition to all these abilities, Lf, as above reported, inhibits the early phase of SARS-CoV (Lang et al., 2011) and SARS-CoV-2 (Hu et al., 2021).

Therefore, based on this information, in order to assess the possibility of using Lf in the clinical COVID-19 treatment, we tested its antiviral activity in *in vitro* experiments to verify whether its activity was associated with the binding of SARS-CoV-2 particles and/or of mammalian cells, similarly to what observed for other viruses (Berlutti et al., 2011; Wakabayashi et al., 2014). Furthermore, the SARS-CoV-2 S trimer structure in prefusion conformation (Wrapp et al., 2020) was used to carry out a protein–protein molecular docking analysis to confirm the hypothesis of a direct interaction between the S glycoprotein and the Lf protein. The structure of the spike glycoprotein (Wrapp et al., 2020) was completed using computational modeling procedures and used to predict Lf interaction sites. Furthermore, the selected high-score protein–protein complex was structurally investigated through classical molecular dynamics (MD) simulation, while the interaction energy between these proteins was evaluated using the molecular mechanics energies combined with generalized Born and surface area continuum solvation (MM/GBSA) method (Genheden and Ryde, 2015).

## Materials and Methods

### *In vitro* Antiviral Activity of Lactoferrin

Lf, extracted from bovine milk (bLf), possesses a high homology of sequence and similar functions with human Lf (hLf), and it has been approved as a generally recognized as safe (GRAS) compound by the United States Food and Drug Administration (FDA United States) and as a dietary supplement by the European Food Safety Authority.

For *in vitro* experiments, highly purified bLf was generously given by Armor Proteines Industries (France). BLf was controlled through SDS-PAGE and silver nitrate staining. BLf purity was about 98%, and its concentration was confirmed via UV spectroscopy according to an extinction coefficient of 15.1 (280 nm, 1% solution). The iron saturation of bLf used, determined via optical spectroscopy at 468 nm, was about 7% according to an extinction coefficient of a 1% solution of bLf completely iron saturated corresponding to 0.54. LPS contamination of bLf, assessed via Limulus Amebocyte assay (Pyrochrome kit, PBI International, Italy), was 0.6 ± 0.05 ng/mg of bLf. Before each *in vitro* assay, bLf solution was sterilized using a 0.2-µm Millex HV filter at low protein retention (Millipore Corp., Bedford, MA, United States).

### Cell Culture and Virus

The African green monkey kidney–derived Vero E6 and human colon carcinoma–derived Caco-2 cells were purchased from American Type Culture Collection (ATCC). Cells were cultivated in high-glucose Dulbecco’s Modified Eagle’s Medium (DMEM) (Euroclone, Milan, Italy) with 10% fetal bovine serum (FBS) (Euroclone, Milan, Italy) at 37°C in humidified incubators with 5% CO_2_. SARS-CoV-2 strain, isolated from nasopharyngeal specimen of a positive COVID-19 patient, was propagated in Vero E6 cells. SARS-CoV-2 titers were obtained by 50% tissue culture infectious dose (TCID50) assays in Vero E6 (Spearman-Kärber method) by microscopic scoring. All assays were performed by infecting Vero E6 and Caco-2 cells with SARS-CoV-2 strain in the Department of Molecular Medicine, University of Padua, under Biosafety Level 3 (BSL3) procedures, in agreement with laboratory containment protocols endorsed by the University of Padua.

### Cytotoxicity Assay

Cytotoxicity was evaluated by incubating 100 and 500 μg of bLf—the concentrations used for *in vitro* experiments—in DMEM with 10% of FBS for 72 h at 37°C with Vero E6 and Caco-2 cells in 96-well plates. Cell viability and proliferation were evaluated by MTT assay (Merck, Italy). The MTT assay is colorimetric assay based on the reduction of a tetrazolium salt to formazan by metabolically active cells. The formazan dye was assessed by spectrophotometric absorbance at 600 nm.

### Infection Assay

For infection assay, Vero E6 cells were seeded in 24-well tissue culture plates at a concentration of 1 × 10^5^ cells/well for 24 h at 37°C in humidified incubators with 5% CO_2_, while Caco-2 cells were seeded at a concentration of 2 × 10^5^ cells/well for 48 h at 37°C in humidified incubators with 5% CO_2_. In order to evaluate the putative inhibition of SARS-CoV-2 strain infection on Vero E6 monkey cells, 100 μg/ml of bLf was used. Conversely, the supposed antiviral activity against SARS-CoV-2 strain on Caco-2 human cells was investigated using not only 100 but also 500 μg/ml of bLf. In order to investigate the putative interaction of bLf with viral particles and/or host cells, different experimental approaches in both Vero E6 and Caco-2 cells were carried out. To evaluate if bLf can interfere with the viral infectivity rate by binding viral surface components, a multiplicity of infection (MOI) of 0.1 and 0.01 of SARS-CoV-2 was preincubated with bLf for 1 h at 37°C in humidified incubators with 5% CO_2_. The cells were then infected with these suspensions for 1 h at 37°C in humidified incubators with 5% CO_2_. In order to evaluate if bLf interferes with the viral attachment to host cells, the cells were preincubated in DMEM without FBS with bLf for 1 h at 37°C in humidified incubators with 5% CO_2_. The cells were then washed with phosphate buffered saline (PBS) and infected with SARS-CoV-2 at an MOI of 0.1 and 0.01 for 1 h at 37°C in humidified incubators with 5% CO_2_. To assess if bLf can interfere with both viral and host cell components, bLf was added together with SARS-CoV-2 at an MOI of 0.1 and 0.01 to cell monolayer for 1 h at 37°C in humidified incubators with 5% CO_2_. In addition, the preincubation of SARS-CoV-2 with bLf for 1 h at 37°C was used to infect cell monolayer previously pretreated with bLf for 1 h at 37°C.

Regarding Vero E6 cells, after each experimental approach, the cells were washed with PBS, covered with DMEM containing 0.75% of carboxymethylcellulose and 2% of FBS and incubated for 48 h at 37°C in humidified incubators with 5% CO_2_. After 48 h, the cells were washed, fixed with 5% of formaldehyde for 10 min at room temperature, and stained with crystal violet at 1% for 5 min. The number of plaques was determined after extensive washing.

The other infection experiments were carried out with Caco-2 cells. Significant cell death was not observed until 7 days on Caco-2 cells after SARS-CoV-2 infection at MOI 0.1 (Chu et al., 2020). In this respect, after each experimental procedure, the cell monolayers were replaced with DMEM with 2% of FBS, and after 6, 24, and 48 h postinfection (hpi), the supernatant samples were collected for RNA extraction and quantitative real-time reverse transcription (rRT)-PCR assay of viral particles. Briefly, we lysed 200 μl of supernatant in an equal volume of NUCLISENS easyMAG lysis buffer (Biomerieux, France). Viral RNA detection was assayed by in-house real-time RT-PCR in accordance with the protocol and the primers and probes designed by Corman et al. (Corman et al., 2020), targeting the genes encoding the SARS-CoV-2 envelope (E) (E_Sarbeco_F, E_Sarbeco_R and E_Sarbeco_P1). Quantitative rRT-PCR analyses were executed with 5 μl of purified nucleic acids in a final volume of 25 μl, employing One-Step Real-Time kit (Thermo Fisher Scientific) and run on ABI 7900HT Fast Sequence Detection Systems (Thermo Fisher Scientific). Cycle threshold (Ct) data from rRT-PCR tests were carried out for E genes. Genome equivalent copies per ml were inferred according to linear regression performed on calibration standard curves.

### Protein–Protein Docking Methods

The SARS-CoV-2 spike glycoprotein structure in prefusion conformation was extracted from a clustering procedure used in a previously published article (Romeo et al., 2020). The 3D structure of the diferric forms of bLf and hLf, refined at 2.8 and 2.2 Å resolution, respectively, were downloaded from the PDB database (PDB IDs: 1BLF (Moore et al., 1997) and 1B0L (Sun et al., 1999)). The protein-protein docking analysis between the modeled SARS-CoV-2 spike glycoprotein (Romeo et al., 2020) and the Lf structures was carried out using the Frodock docking algorithm (Ramírez-Aportela et al., 2016). Frodock’s approach combines the projection of the interaction terms into 3D grid-based potentials and the binding energy upon complex formation, which is approximated as a correlation function composed of van der Waals, electrostatics, and desolvation potential terms. The interaction-energy minima are identified through a fast and exhaustive rotational docking search combined with a simple translational scanning (Garzon et al., 2009). Both docking procedures were performed using Frodock’s (http://frodock.chaconlab.org/) web-server.

### Molecular Dynamics

Topology and coordinate files of the input structures have been obtained through the tLeap module of the AmberTools 19 package (Salomon-Ferrer et al., 2013). The spike glycoprotein and Lf were parametrized through the ff19SB force field and were inserted into a rectangular box of TIP3P water molecules, imposing a minimum distance of 12.0 Å from the box walls, while the solution was neutralized adding 0.15 mol/L of NaCl ions. To remove steric interactions, all structures underwent four minimization cycles, each composed by 500 steps of steepest descent minimization followed by 1,500 steps of conjugated gradient minimization. An initial restraint of 20.0 kcal mol^−1^ Å^−2^ was imposed on protein atoms and subsequently reduced and removed in the final minimization cycle. Systems were gradually heated from 0 to 300 K in an NVT ensemble over a period of 2.0 ns using the Langevin thermostat, imposing a starting restraint of 0.5 kcal mol^−1^ Å^−2^ on each atom, which was decreased every 500 ps in order to slowly relax the system. The systems were simulated in an isobaric-isothermal (NPT) ensemble for 2.0 ns, fixing a pressure of 1.0 atm using the Langevin barostat and imposing the temperature at 300 K. Covalent bonds involving hydrogen atoms were constrained using the SHAKE algorithm (Ryckaert et al., 1977). 30 ns of production run were performed through the NAMD 2.13 MD package (Phillips et al., 2005), using a time step of 2.0 fs. The PME method was applied to take into account long-range interactions, while a cutoff of 9.0 Å was set for short-range interactions. System coordinates were saved every 1,000 steps.

### Trajectory Analysis

Distance analysis was carried out using the distance module of the GROMACS 2020 analysis tools (Abraham et al., 2015), while hydrogen bond persistence was evaluated using the hbonds module coupled to in-house written codes. The hydrophobic contacts were identified using the contact_map and contact_frequency routines of the mdtraj Python library (McGibbon et al., 2015). Generalized Born and surface area continuum solvation (MM/GBSA) analyses were carried out using the last 15 ns of the trajectories, through the MMPBSA. py.MPI program as implemented in the AMBER16 software (Case et al., 2016) on two nodes of the ENEA HPC cluster CRESCO6 (Iannone et al., 2019). Pictures of the spike-Lf and spike CTD1-ACE2 complexes were generated using the UCSF Chimera program (Pettersen et al., 2004).

### Statistical Analysis

For *in vitro* experiments, the number of plaque-forming units (pfu)/ml of SARS-CoV-2 on Vero E6 cells and the number of SARS-CoV-2 RNA copies/ml on Caco-2 cells in each experimental approach was compared to the control ones (untreated SARS-CoV-2 and cells) at the same time point in order to assess the statistically significant differences by using unpaired student’s *t* tests. Results were expressed as the mean values ± standard deviation (SD) of three independent experiments. In each case, a *p* value ≤0.05 was considered statistically significant.

## Results

### Lactoferrin Displays Antiviral Properties in *In Vitro* Models

Preliminarily, the doses of bLf in native form (7% iron saturated) corresponding to 100 μg/ml for Vero E6 cells and 100 and 500 μg/ml for Caco-2 cells were assayed to detect their putative cytotoxicity by measuring cell morphology, proliferation, and viability after 72 h of incubation. Both 100 and 500 μg/ml of bLf did not exert any cytotoxic effect (data not shown).

Then, the efficacy of different concentrations of bLf in the inhibition of SARS-CoV-2 infection was tested on Vero E6 and Caco-2 cells according to different experimental procedures: I) control: untreated SARS-CoV-2 and cells; II) preincubation of bLf with virus inoculum for 1 h at 37°C before cell infection; III) preincubation of bLf with cells for 1 h at 37°C before virus infection; IV) bLf added together with SARS-CoV-2 at the moment of infection; and V) virus and cells separately preincubated with bLf for 1 h at 37°C before infection.

**FIGURE 1:**
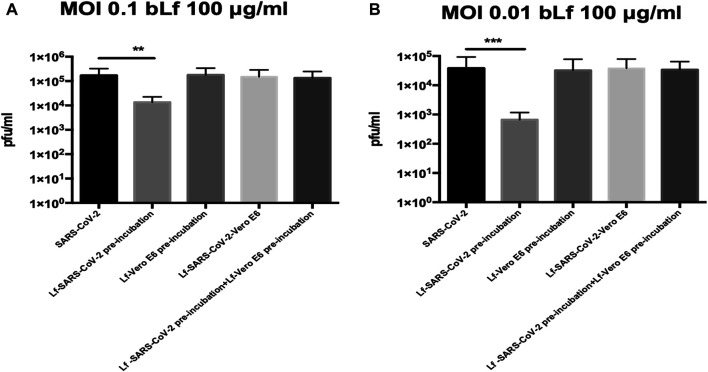
Plaque-forming units (pfu)/ml of SARS-CoV-2 observed in Vero E6 cells infected at multiplicity of infection (MOI) of 0.1 **(A)** and 0.01 **(B)** in the presence or absence of 100 μg/ml of bovine lactoferrin (bLf) according to the following experimental procedures: i) control: untreated SARS-CoV-2 and Vero E6 cells; ii) bLf preincubated with SARS-CoV-2 inoculum for 1 h at 37°C before cell infection; iii) cells preincubated with bLf for 1 h at 37°C before SARS-CoV-2 infection; iv) bLf added together with SARS-CoV-2 inoculum during the adsorption step; and v) virus and cells separately preincubated with bLf for 1 h at 37°C before infection. Data represent the mean values of three independent experiments. Error bars: standard error of the mean. Statistical significance is indicated as follows: **: *p* < 0.001, ***: *p* < 0.0001 (unpaired student’s *t* test).

The results obtained with Vero E6 cells are shown in Figure 1A (MOI 0.1) and 1B (MOI 0.01).

Regarding Vero E6 cells, an inhibition of SARS-CoV-2 replication of about one log at MOI 0.1 and about two log at MOI 0.01 was observed when 100 μg/ml of bLf was preincubated for 1 h with virus before infection compared to untreated SARS-CoV-2 infection (*p* < 0.001 and *p* < 0.0001, respectively) (Figures 1A,B).

On the contrary, the data illustrated in Figures 1A,B, independently from the MOI used, indicate that bLf, at this concentration, does not block SARS-CoV-2 infection when it is preincubated with Vero E6 cells or when bLf is contemporary added to viral particles and cells at the moment of infection (Figures 1A,B). BLf is also ineffective when it is preincubated for 1 h at 37°C separately with virus and cells before infection (Figures 1A,B).

**FIGURE 2:**
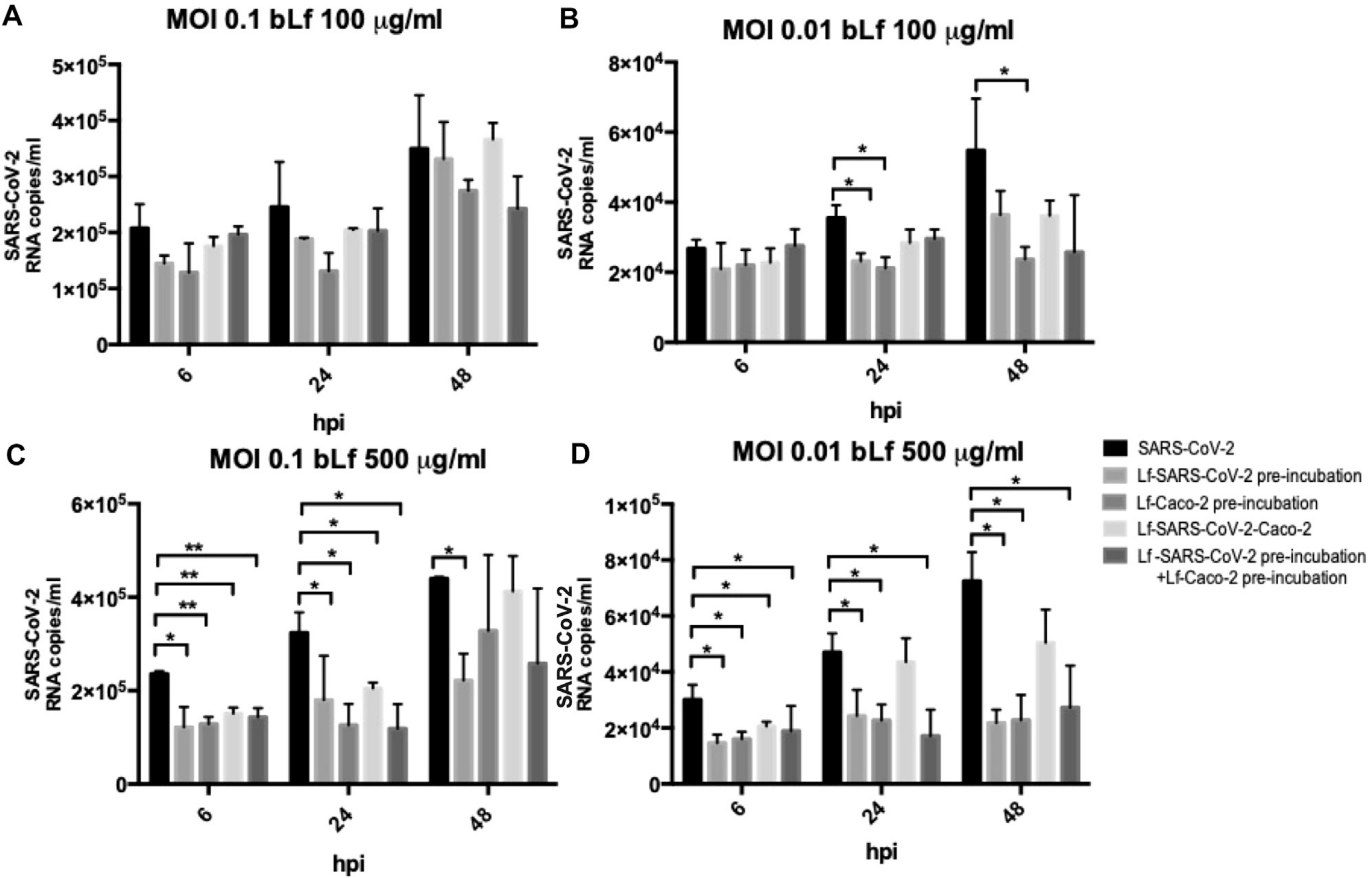
RNA copies/ml of SARS-CoV-2 observed in supernatants of Caco-2 cells infected at multiplicity of infection (MOI) of 0.1 **(A,C)** and 0.01 **(B,D)** in the presence or absence of 100 μg/ml **(A,B)** and 500 μg/ml **(C,D)** of bovine lactoferrin (bLf) according to the following experimental procedures: i) control: untreated SARS-CoV-2 and Caco-2 cells; ii) bLf preincubated with SARS-CoV-2 inoculum for 1 h at 37°C before cell infection; iii) cells preincubated with bLf for 1 h at 37°C before SARS-CoV-2 infection; iv) bLf added together with SARS-CoV-2 inoculum during the adsorption step; and v) virus and cells separately preincubated with bLf for 1 h at 37°C before infection. Viral supernatant samples were harvested at 6, 24, and 48 hours postinfection (hpi). Viral loads were ascertained with quantitative rRT-PCR. Data represent the mean values of three independent experiments. Error bars: standard error of the mean. Statistical significance is indicated as follows: *: *p* < 0.05, **: *p* < 0.001 (Unpaired student’s *t* test).

The efficacy of 100 and 500 μg/ml of bLf against SARS-CoV-2, assayed in Caco-2 cells, is showed in Figures 2A,B (MOI 0.1) and in Figures 2C,D (MOI 0.01), respectively.

Regarding Caco-2 cells, at MOI 0.1, no significant differences were observed in all experimental conditions compared to the control ones when using bLf at 100 μg/ml (Figure 2A). At MOI 0.01, an inhibition of viral load in supernatants was observed at 24 hpi only when 100 μg/ml of bLf was preincubated with the viral inoculum and when the cells were preincubated with 100 μg/ml of bLf compared to the control one (*p* < 0.05) (Figure 2B). At 48 hpi, an inhibition of viral load was observed only when the cells were preincubated with bLf (*p* < 0.05) (Figure 2B).

When bLf was used at a concentration of 500 μg/ml, a decrease of viral load up to 48 hpi was observed when the viral inoculum was preincubated with bLf compared to the control group, independently from the MOI used (*p* < 0.05) (Figures 2C,D). When the cells were preincubated with bLf, a decrease of SARS-CoV-2 load up to 24 hpi was observed compared to the control at MOI 0.1 (*p* < 0.001 after 6 hpi and *p* < 0.05 after 24 hpi) (Figure 2C), while at MOI 0.01 the decrease of viral load remained statistically significant up to 48 hpi compared to the control group (*p* < 0.05) (Figure 2D). When bLf was added together with SARS-CoV-2 during the adsorption step a decrease of viral load up to 24 hpi was observed compared to untreated SARS-CoV-2 infection, independently from the MOI used (*p* < 0.001 after 6 hpi and *p* < 0.05 after 24 hpi for MOI 0.1; *p* < 0.05 after 6 and 24 hpi for MOI 0.01) (Figures 2C,D). When the cells were preincubated with bLf and infected with SARS-CoV-2 previously preincubated with bLf, a decrease of viral load up to 24 hpi was observed for MOI 0.1 compared to untreated SARS-CoV-2 infection (*p* < 0.001 after 6 hpi and *p* < 0.05 after 24 hpi for MOI 0.1) (Figure 2C), while at MOI 0.01 the decrease of viral load remained statistically significant up to 48 hpi compared to untreated SARS-CoV-2 infection (*p* < 0.05) (Figure 2D).

### Computational Results

**FIGURE 3:**
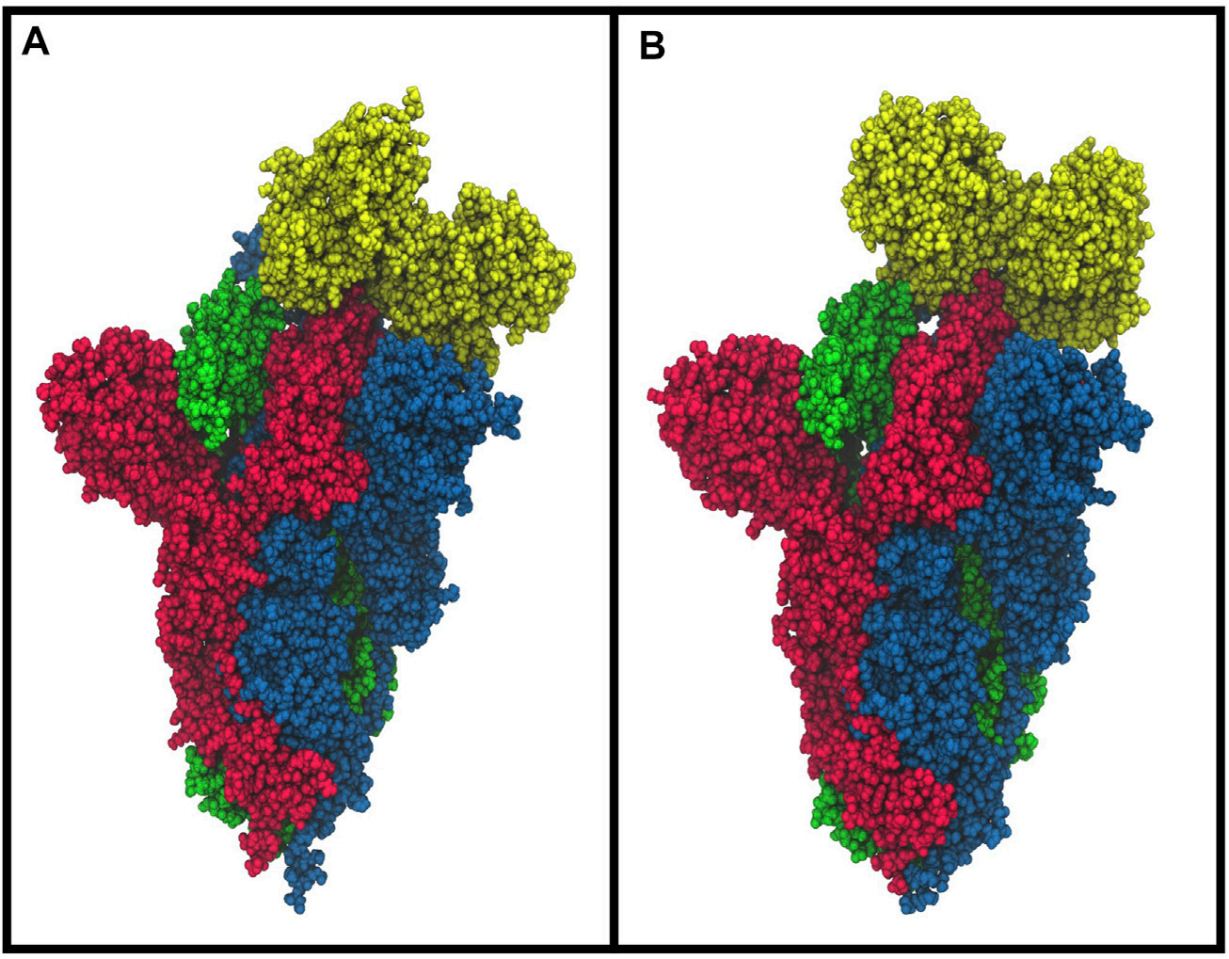
Space-fill representations of the best molecular complex obtained with Frodock between the bovine **(A)** and human **(B)** lactoferrin with the spike glycoprotein. The red, blue, and green colors represent the spike glycoprotein chains, and while yellow depicts the lactoferrin molecules.

The molecular docking simulation suggests a potential interaction of the bLf structure with the spike glycoprotein CTD1 domain in the up conformation (Figure 3A). The first three solutions obtained by Frodock clustering procedure account for more than 60% of the total generated complexes and are almost completely superimposable to that shown in Figure 3A. Starting from the first Frodock solution, we performed a 30 ns long classical MD simulation in order to verify the stability of the complex and check for the presence of persistent interactions between the two proteins. As shown in Supplementary Figure S1A, the distance between the centers of mass of spike and bLf, calculated as a function of time, oscillates around the value of 4.5 nm, indicating a constant close contact between the two molecules. MM/GBSA analysis confirmed the high affinity of the bLf for the spike CTD1 domain (Supplementary Table S1A), showing interaction energy of −28.02 kcal/mol. In particular, MM/GBSA results underlined that the Van der Waals term mainly contribute to the binding energy (Supplementary Table S1A).

A detailed analysis of the interaction network revealed the presence of 28 different interactions, which persist for more than 25% of the simulation time, in agreement with the high interaction energy calculated. In detail, we found three salt bridges, five hydrogen bonds and 20 residue pairs involved in hydrophobic contacts (Supplementary Table S2 left side).

**FIGURE 4:**
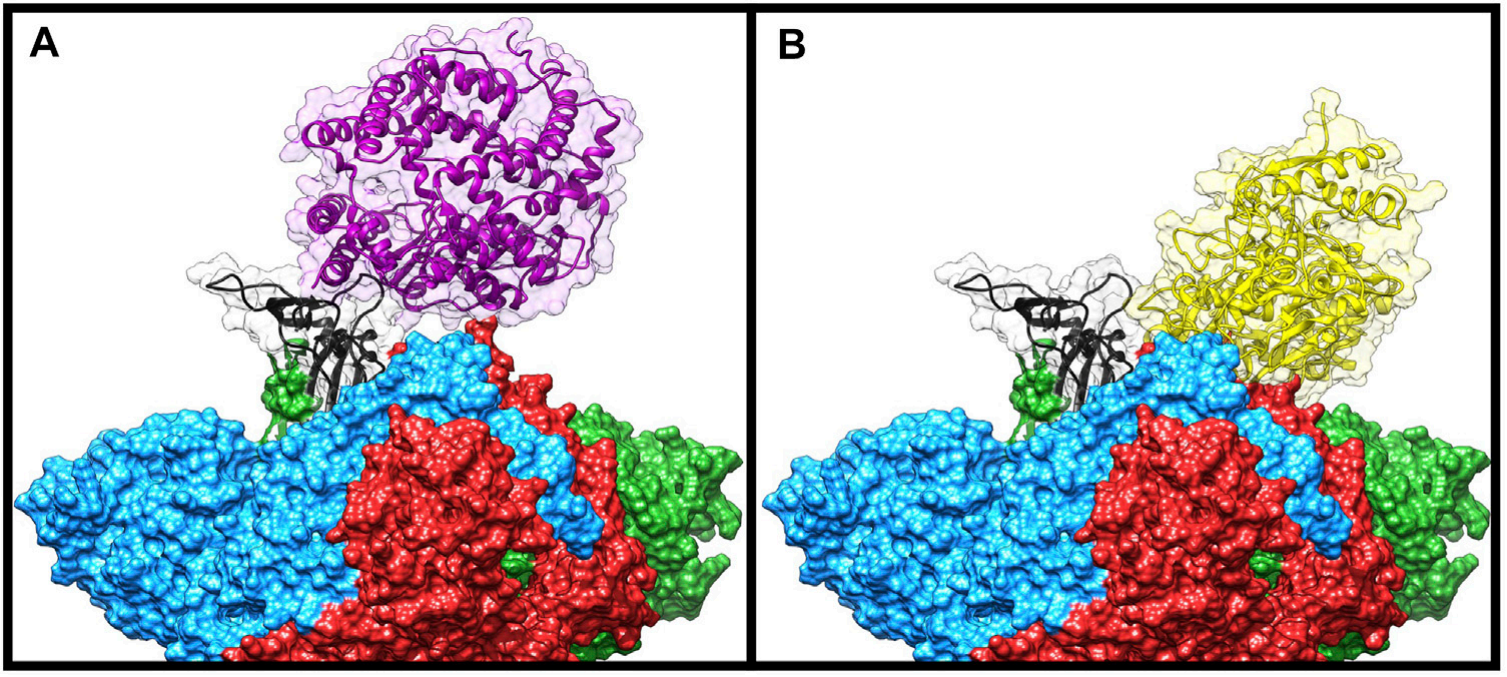
Comparison of the Frodock best complex and ACE2-spike glycoprotein (PDB ID: 6LZG). The red, blue, and green solid surfaces represent the three different chains composing the spike glycoprotein. The black ribbons highlight the CTD1 domain in the up conformation. The magenta and yellow ribbons represent the ACE2 **(A)** and the bovine lactoferrin **(B)**, respectively, surrounded by a transparent molecular surface representation, in order to point out the positions occupied in the space by the different structures.

To check if some of the spike residues targeted by the bLf protein were involved in the binding with ACE2, we compared the average structure extracted from the simulation with the ACE2/CTD1 domain complex structure (PDB ID: 6LZG (Wang et al., 2020)) (Figure 4). Surprisingly, only two spike residues (Gly502 and Tyr505) were shared between the complexes interfaces (Supplementary Table S2 left side), as evaluated from the inspection of the superimposed structures and from the article analysis (Wang et al., 2020). Despite this, Lf holds the same position assumed by the ACE2 enzyme, that is, above the up CTD1 domain.

We performed the same analysis over the evaluated hLf–spike complex, obtaining a binding pose superimposable to that observed for the bovine protein (Figure 3B). Although using the human protein we could still observe a persistent and close contact between the two molecules (Supplementary Figure S1B), the analysis of the interaction network indicated the presence of a larger number of interactions (45 interactions), in agreement with the higher interaction energy revealed by the MM/GBSA approach (−48.25 kcal/mol, Supplementary Table S1B). In detail, we found 12 salt bridges, 10 hydrogen bonds and 23 residue pairs involved in hydrophobic contacts (Supplementary Table S2 right side), in agreement with the presence of a negative electrostatic contribution term (Supplementary Table S1B). Comparing the average structure extracted from the simulation with the ACE2/CTD1 domain complex structure (PDB ID: 6LZG (Wang et al., 2020)) (Supplementary Figure S2), we observed that also for the hLf, only two residues (Thr500 and Tyr505) were shared between the complexes interfaces (Supplementary Table S2 right side).

These results allow us to hypothesize that, in addition to the HSPGs binding (Lang et al., 2011), both bLf and hLf should be able to hinder the spike glycoprotein recognition of the ACE2 receptor, blocking the virus from entering into the cells.

## Discussion

In this study, we focused our attention on the well-known antiviral activity of Lf. The *in vitro* antiviral activity of bLf against enveloped and naked DNA and RNA viruses has been widely demonstrated (van der Strate et al., 2001; Berlutti et al., 2011; Lang et al., 2011; Wakabayashi et al., 2014; Ng et al., 2015), while some articles have been published on its *in vivo* efficacy against viral infection (Lu et al., 1987; Tanaka et al., 1999; Okada et al., 2002; Hirashima et al., 2004; Ishibashi et al., 2005; Shin et al., 2005; Ueno et al., 2006; Egashira et al., 2007; Chen et al., 2008; Yen et al., 2011; Gualdi et al., 2013; Vitetta et al., 2013).

The capability of bLf to hinder viral infection is generally attributed to its binding to cell surface anionic components and/or viral particles. BLf is able to competitively bind to heparan sulfate proteoglycans (HSPGs), components of the host cell surface and identified as initial interaction sites for enveloped viruses (Spear, 2004; Sapp and Bienkowska-Haba, 2009), thus hindering the adhesion and internalization of several viruses (Marchetti et al., 2004; Chien et al., 2008; Lang et al., 2011), including SARS-CoV-2 (Hu et al., 2021). Moreover, bLf can also bind directly to surface proteins of virus particles as HIV V3 loop of the gp120 (Swart et al., 1996) and HCV E2 envelope proteins (Nozaki et al., 2003).

The results presented here show that the antiviral activity of bLf varies according to different experimental approaches, cell lines, MOI, and bLf concentrations.

As a matter of fact, the preincubation of 100 μg/ml of bLf with Vero E6 monolayers, infected with SARS-CoV-2 at MOI 0.1 and 0.01, was ineffective in inhibiting virus internalization (Figure 1), differently from what observed in Caco-2 cells at MOI 0.01 (Figure 2B).

The preincubation of 100 μg/ml of bLf with SARS-CoV-2 showed a significantly higher antiviral activity at MOI 0.01 than MOI 0.1 in Vero E6 cells (Figures 1A,B), while a significant antiviral activity was observed only at MOI 0.01 in Caco-2 cells (Figure 2B). In the other two experimental conditions, 100 μg/ml of bLf did not show any significant antiviral activity on both Vero E6 and Caco-2 cells (Figures 1, 2A,B).

Differently from 100 μg/ml of bLf, the preincubation of 500 μg/ml of bLf with Caco-2 cells or viral particles showed a higher decrease in the viral load at MOI 0.1 and 0.01 (Figures 2C,D). In the other two experimental approaches, 500 μg/ml of bLf was significantly effective against SARS-CoV-2, even if for different times of postinfection and at different extents depending on MOI (Figures 2C,D).

Our experimental results indicate that bLf exerts its antiviral activity either by direct binding to the SARS-CoV-2 particles or by obscuring their host cell receptors. Moreover, the results obtained through the molecular docking and molecular dynamics simulation approaches strongly support the hypothesis of a direct recognition between the bLf and the spike glycoprotein. The affinity between their molecular surfaces, the large number of atomistic interactions detected and their persistence during the simulation suggest that this recognition is very likely to occur and that bLf may hinder the spike binding to the ACE2 receptor, thus blocking virus entry into host cells.

Taken together, these results reveal that, even if the definitive mechanism of action still has to be completely investigated, the antiviral properties of bLf are also extendable to SARS-CoV-2 virus.

This study is part of the GEFACOVID2.0 research program coordinated by the Tor Vergata University of Rome.

## Data Availability

The original contributions presented in the study are included in the article/Supplementary Material, and further inquiries can be directed to the corresponding author.
