# Supplementary material for: Lactoferrin Against SARS-CoV-2: In Vitro and In Silico Evidences
Source: Front Pharmacol. 2021 Jun 17;12:666600. doi: 10.3389/fphar.2021.666600 (PMC8242182; doi:10.3389/fphar.2021.666600)
Supplement: Supplementary file 1 [file DataSheet1.PDF]

Supplemental data

Figures

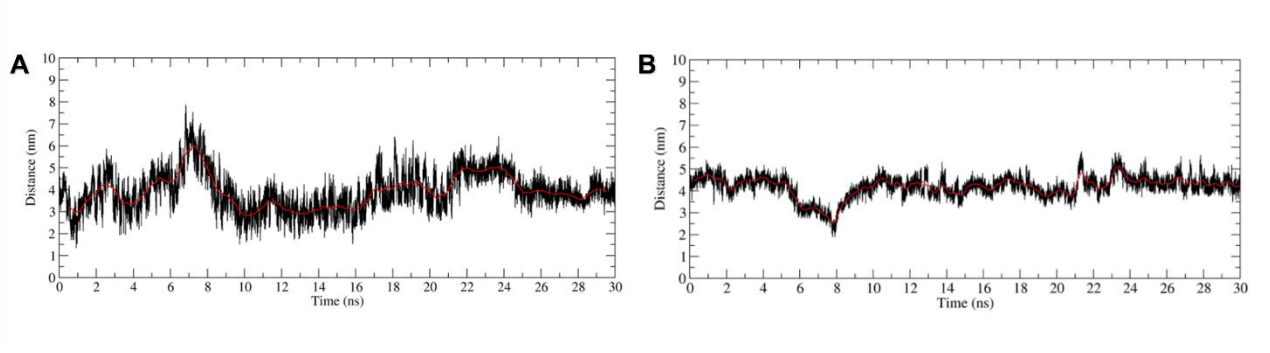

Figure S1. A) Time-dependent analysis of the distance evaluated between the centers of mass of the CTD1 domain in the up conformation and of the bovine lactoferrin. The red line represents the distance values averaged over 250 trajectory frames. **B)** Time-dependent analysis of the distance evaluated between the centers of mass of the CTD1 domain in the up conformation and of the human lactoferrin. The red line represents the distance values averaged over 250 trajectory frames.

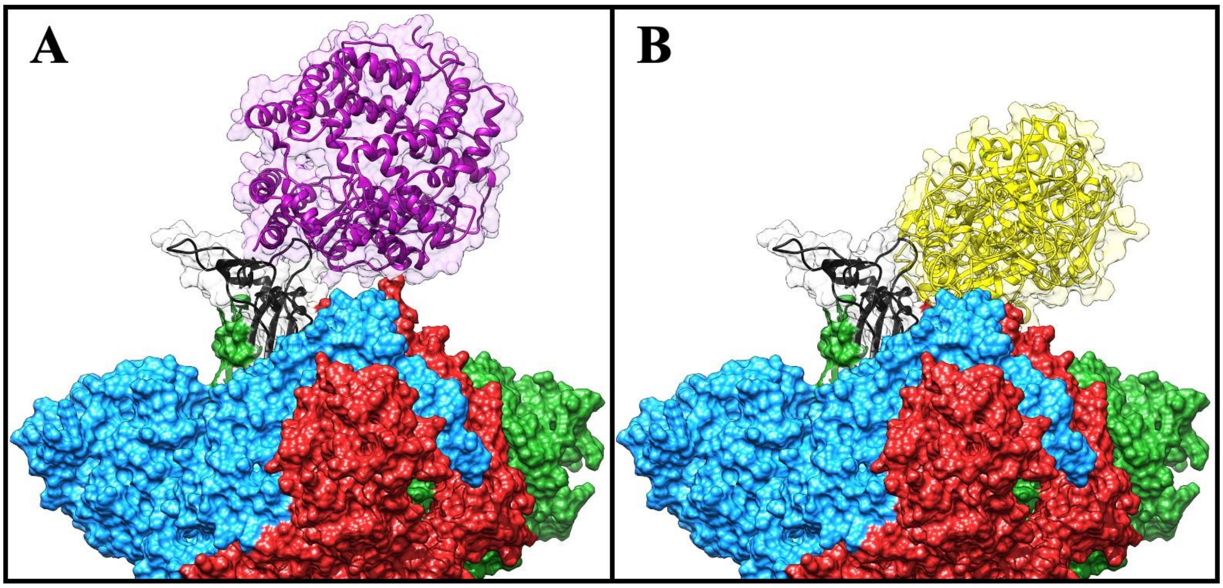

Figure S2. Structural comparison of the Frodock best complex and of the ACE2-Spike glycoprotein (PDB ID: 6LZG). The red, blue and green solid surfaces represent the three different chains composing the Spike glycoprotein. The black ribbons highlight the CTD1 domain in the up conformation. The magenta and yellow ribbons represent the ACE2. **(A)** and the human lactoferrin **(B)**, respectively, surrounded by a transparent molecular surface representation, in order to point out the positions occupied in the space by the different structures.

Tables

A) Spike-bovine lactoferrin complex simulation				
**VdW (kcal/ mol)**	**Electrostatic (kcal/mol)**	**Nonpolar solvation (kcal/ mol)**	**Polar solvation (kcal/ mol)**	**Interaction energy (kcal/ mol)**
-138.27	125.11	-42.29	-18.35	-28.02
**B) Spike-human lactoferrin complex simulation**				
**VdW (kcal/ mol)**	**Electrostatic (kcal/mol)**	**Nonpolar solvation (kcal/ mol)**	**Polar solvation (kcal/ mol)**	**Interaction energy (kcal/ mol)**
-158.27	-115.80	-21.18	209.78	-48.25

Table S1. A) Results of the MM/GBSA analyses performed over the last 15 ns of the Spike-bovine lactoferrin complex simulation**. B)** Results of the MM/GBSA analyses performed over the last 15 ns of the Spike-human lactoferrin complex simulation.

**Interaction (Spike - bovine lactoferrin)**	**Interaction type**	**Interaction (Spike – human Lactoferrin)**	**Interaction type**
Glu159-Arg408	salt bridge	Lys378-Glu356	salt bridge
Glu162-Lys417	salt bridge	Asp405-Arg362	salt bridge
Glu355-Arg408	salt bridge	Asp405-Arg628	salt bridge
		Glu406-Arg362	salt bridge
Gly404-Glu355	non-polar contact	Glu406-Arg628	salt bridge
Asp405-Glu355	non-polar contact	Arg408-Glu355	salt bridge
Arg408-Ala354	non-polar contact	Arg408-Glu356	salt bridge
Arg408-Glu355	non-polar contact	Lys444-Glu128	salt bridge
Arg408-Lys358	non-polar contact	Lys462-Asp449	salt bridge
Arg408-Thr353	non-polar contact	Glu465-Hie635	salt bridge
Asn437-Gln386	non-polar contact	Glu465-Lys639	salt bridge
Asn439-Gln386	non-polar contact	Arg466-Asp646	salt bridge
Gly502-Glu356	non-polar contact		
Gly502-Thr353	non-polar contact	Arg403-Arg362	non-polar contact
Val503-Ala359	non-polar contact	Asp405-Arg362	non-polar contact
Val503-Arg363	non-polar contact	Arg408-Arg362	non-polar contact
Val503-Glu352	non-polar contact	Asn437-Asn157	non-polar contact
Val503-Glu355	non-polar contact	Asn439-Asn127	non-polar contact
Val503-Glu356	non-polar contact	Asn439-Pro154	non-polar contact
Val503-Thr353	non-polar contact	Ser443-Asn127	non-polar contact
Gly504-Glu355	non-polar contact	Ser443-Asn254	non-polar contact
Gly504-Glu356	non-polar contact	Ser443-Phe155	non-polar contact
Tyr505-Gln386	non-polar contact	Asn440-Gln130	non-polar contact
Gln506-Gln386	non-polar contact	Lys444-Asn127	non-polar contact
		Lys444-Glu128	non-polar contact
Asp405-Ser160	hydrogen bond	Pro499-Asn254	non-polar contact
Arg434-Glu355	hydrogen bond	Pro499-Asp253	non-polar contact
Thr526-Ser160	hydrogen bond	Pro499-Leu126	non-polar contact
Asn527-Ser160	hydrogen bond	Pro499-Phe155	non-polar contact
Val529-Ser160	hydrogen bond	Pro499-Pro252	non-polar contact
		Thr500-Asp253	non-polar contact
		Thr500-Pro252	non-polar contact
		Val503-Arg362	non-polar contact
		Gly504-Arg362	non-polar contact
		Tyr505-Pro154	non-polar contact
		Tyr508-Thr159	non-polar contact
		Asp405-Arg362	hydrogen bond
		Arg408-Glu356	hydrogen bond
		Asn437-Asn157	hydrogen bond
		Asn437-Pro154	hydrogen bond
		Asn439-Pro154	hydrogen bond
		Asn440-Gln130	hydrogen bond
		Ser443-Asn127	hydrogen bond
		Lys444-Asn127	hydrogen bond
		Lys444-Glu128	hydrogen bond
		Thr500-Pro252	hydrogen bond

Table S2 (left side) Molecular interactions established between the CTD1 domain of the Spike protein and the bovine lactoferrin. Only interactions identified for more than 50% of simulation time are shown. Residues highlighted in grey are shared in the interfaces of Spike-ACE2 and Spike-bovine lactoferrin. **(right side)** Molecular interactions established between the CTD1 domain of the Spike protein and the human lactoferrin. Only interactions identified for more than 50% of simulation time have been showed. Residues highlighted in grey are shared in the interfaces of Spike-ACE2 and Spike-human lactoferrin.
